# The Cherokee County Medical Society

**Published:** 1891-03

**Authors:** J. K. Frazer

**Affiliations:** Secretary


The Cherokee County Medical Association held its regular meeting in January with its usual interest. There being no response from the Committees on Collective Investigation, Dr. Smith reported a case of congestion—a child at ten years successfully treated with quinine and tincture of belladona. This case was given instead of a regular paper, and created more discussion than any other, as to the use and doses of quinine during the poroxysm.

Dr. Wm. Osborne read a paper entitled "Diagnosis of a few Conditions of the Blood, Stomach and Intestines, from the Appearance of the Tongue."

The Doctor thinks from the unanimity in making this examination, it should be regarded as one of the most important means of diagnosis, if properly understood. The paper was tolerably exhaustive, gave the different appearances of the tongue in the different stages of disease, and what the appearances indicated, with suggestions as to the proper remedial agent to be used. The conclusions of which he thinks we may safely classify the appearance of the tongue as having reference, 1st, to the condition of the blood; 2nd, as indicating the condition of the digestive organs. The paper was carefully prepared, and this, with Dr. Smith's paper, was received by the Association and discussed by Drs. Brittain, Guinn and Evans, also Guinn, Rusk and others. We had, as we usually do, an interesting convocation. The wonder is why *all* who can do not attend.

The officers elected for the ensuing year are: Dr. A. H. McCord, President; Drs. H. V. Collins, J. N. B. Guinn and R. W. Tennison, Vice-Presidents; J. K. Frazer, Secretary; J. H. Evans, Assistant Secretary.

For our next meeting we have papers on—

"Therapeutics of Binoxide of Hydrogen," by Dr. J. H. Evans.

"Acute Yellow Atrophy of the Liver," by Dr. W. G. Jameson.

"Black Jaundice," by Dr. A. W. Barton.

"Treatment of Phthisis," by Dr. N. W. Spring.

The meeting adjourned to meet at Alto on the first Thursday in April, when, it is hoped, all will attend who can.

J. K. FRAZER, Secretary.

